# Decoration of Zinc Oxide Nanorods into the Surface of Activated Carbon Obtained from Agricultural Waste for Effective Removal of Methylene Blue Dye

**DOI:** 10.3390/ma13245667

**Published:** 2020-12-11

**Authors:** Priyanka Shrestha, Manoj Kumar Jha, Jeevan Ghimire, Agni Raj Koirala, Rajeshwar Man Shrestha, Ram Kumar Sharma, Bishweshwar Pant, Mira Park, Hem Raj Pant

**Affiliations:** 1 Nanomaterials Lab, Department of Applied Sciences and Chemical Engineering, Pulchowk Campus, Institute of Engineering, Tribhuvan University, Kathmandu 44600, Nepal; shrestha.pri@gmail.com (P.S.); jhamanoj144@gmail.com (M.K.J.); rejeevanghimi7@gmail.com (J.G.); rmschemrhos@gmail.com (R.M.S.); rksharma2002@ioe.edu.np (R.K.S.); 2 Korea Center for Artificial Photosynthesis, Department of Chemistry, Sogang University, Shinsu-dong, Mapo-go, Seoul 121-742, Korea; agnikoirala@gmail.com; 3 Carbon Composite Energy Nanomaterials Research Center, Woosuk University, Wanju-Gun, Jeollabuk-do 55338, Korea

**Keywords:** ZnO nanorods, activated carbon, composite, hydrothermal, photocatalyst

## Abstract

Zinc oxide (ZnO) nanorods incorporated activated carbon (AC) composite photocatalyst was synthesized using a hydrothermal process. The AC was prepared from lapsi (*Choerospondias axillaris*) seed stone, an agricultural waste product, found in Nepal by the chemical activation method. An aqueous suspension of AC with ZnO precursor was subjected to the hydrothermal treatment at 140 °C for 2 h to decorate ZnO rods into the surface of AC. As-obtained ZnO nanorods decorated activated carbon (ZnO/AC) photocatalyst was characterized by various techniques, such as scanning electron microscopy (SEM), X-ray diffraction (XRD), and photoluminescence (PL) spectroscopy. Results showed that highly crystalline hexagonal ZnO nanorods were effectively grown on the surface of porous AC. The photocatalytic property of the as-prepared ZnO/AC composite was studied by degrading methylene blue (MB) dye under UV-light irradiation. The ZnO/AC composite showed better photocatalytic property than that of the pristine ZnO nanorods. The enhanced photocatalytic performance in the case of the ZnO/AC composite is attributed to the combined effects of ZnO nanorods and AC.

## 1. Introduction

Dyes in the water body are the effluents from textile industries which are colored, toxic, and carcinogenic to living organisms [1,2,3]. They decrease the dissolved oxygen levels and increase the chemical oxygen demand in water and create an unfavorable condition for aquatic lives. Different studies have shown that such effluents can be removed by using adsorptive or photocatalytic materials. Therefore, designing effective adsorbent/photocatalyst is essential to meet the demand of removing dye wastes created by textile industries. In recent years, various metal oxide-based semiconductors such as TiO_2_, ZnO, Fe_2_O_3_, CuO, RuO_2_, NiO, CeO, Cr_2_O_3_, [1,4,5,6,7] etc., have been considered as an effective photocatalyst for wastewater treatment. Among them, zinc oxide (ZnO) is inexpensive, abundantly found, biocompatible, and a physically and chemically stable photocatalyst [8,9]. Although ZnO is the leading candidate in photocatalysis [10,11], low photon-to-electron conversion efficiency, high electron-hole recombination rate, and poor adsorption capacity are the major limitations for practical application [11,12,13]. Therefore, for the effective implementation of ZnO in photocatalysis, these limitations should be overcome by improved designs and synthetic protocols.

Synthesizing nano-sized particles could be an effective strategy to enhance the photocatalytic property of ZnO owing to their high surface area [14]; however, the nano-sized particles may cause secondary pollution due to the difficulties in recovery in the reaction system [15,16]. In addition, the nanostructured photocatalysts have a tendency to agglomerate, which is another disadvantage in photocatalysis [17,18]. Immobilization of photocatalyst nanoparticles into the carbon source could be considered an effective strategy to solve the aforementioned issues. For example, recently, our group has developed various metal oxides nanoparticles incorporated carbon nanofibers photocatalysts with good photocatalytic property and reusability [15,19,20,21,22]. In the carbon-supported photocatalytic system, the carbon fiber provides support for the deposition of photocatalytic materials as well as acts as a good adsorbent, leading to the overall enhancement in the photocatalytic performance of the composite material [23,24].

Among the various forms of carbon, the activated carbons (ACs) have been considered for the immobilization of photocatalytic materials due to their favorable properties such as large surface area, porosity, good adsorptivity, and convenience in handling [25,26,27]. The composites of ZnO with ACs have been synthesized in previous studies [28,29,30]; however, the high cost of commercial AC limits its application worldwide [2]. Therefore, there is a growing interest in the economic production of ACs. In recent years, the use of low-cost wastes and agricultural by-products has gained great attention to produce porous AC economically. Different agricultural waste materials including peach stones, apricot stones, lapsi seed stone, black stone cherries, guava seeds, orange peel, sugarcane bagasse, peanut shell, and many more have been reported to produce AC [31,32,33,34]. Among the various agro-based wastes, the hard lapsi (*Choerospondias axillaris*) seed stone (locally found in Nepal) can be considered as an excellent source of AC. The AC with good porosity has been prepared from lapsi seed stone by using phosphoric acid as an activating agent [35].

In this study, we tried to exploit the photocatalytic property of ZnO and the adsorption property of AC from lapsi seed stone by incorporating ZnO nanorods into the AC using the hydrothermal technique. The functionalities present on AC due to the phosphoric acid activation can provide a nucleation site for the growth of ZnO NPs over the surface of porous AC during the hydrothermal treatment, leading to the formation of the ZnO decorated activated carbon (ZnO/AC) composite. In the ZnO/AC composite photocatalyst, the conductive nature of AC can also help in the charge separation, which can enhance the overall photocatalytic activity of the ZnO/AC composite. In addition to this, the proper incorporation of ZnO nanorods into AC prevents the loss of photocatalyst during recovery.

## 2. Materials and Methods

### 2.1. Materials

The lapsi fruits (*Choerospondias axillaris*) were purchased from the local market (Kalimati) of Kathmandu, Nepal. Zinc nitrate hexahydrate, phosphoric acid, methylene blue (MB) were purchased from Sigma-Aldrich (Saint Louis, MO, USA). Bis-hexamethylenetetramine was obtained from the Samchun Chemicals, Seoul, Korea. Ethanol was obtained from the Daejung Chemicals, Shiheung-city, Gyeonggi-do, Korea. All chemicals were used without further purification.

### 2.2. Synthesis of AC from the Lapsi Seed

The fruits were boiled to separate stones from soft material. The seed stones were washed several times with distilled water and alcohol to remove soft impurities. After drying at 110 °C for 12 h, the seed stones were crushed with a mortar and electric grinder followed by sieving as reported in our previous study [24]. Next, AC was prepared by chemical activation with 50% phosphoric acid (1:1 weight ratio) and carbonization for 4 h at 800 °C in a horizontal tubular furnace under a flow of nitrogen gas (75 mL/min). The product was washed with NaHCO_3_ solution, followed by frequent washing with distilled water. The as-obtained AC powder was kept in an oven at 110 °C for 12 h. The final product was the mesoporous AC with carbon yield about 9.3%.

### 2.3. Synthesis of ZnO/AC Composite Photocatalyst

**Figure 1:**
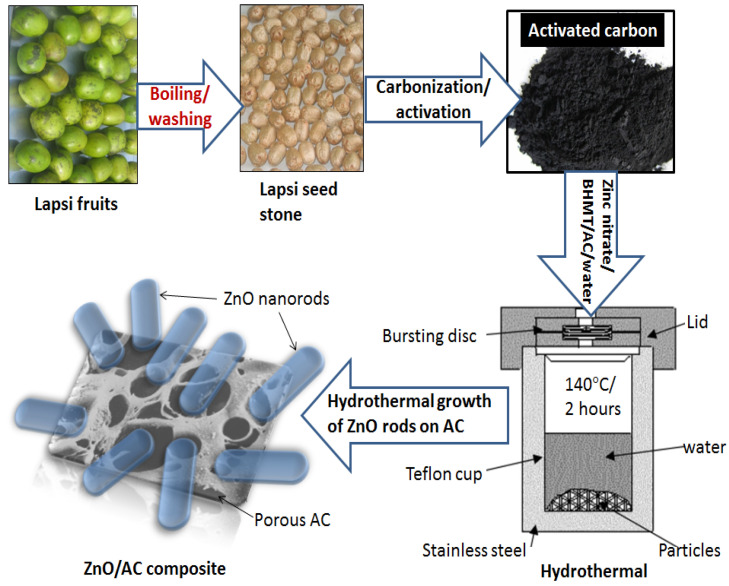
Schematic diagram showing the preparation of ZnO/AC composite photocatalyst.

In the beginning, 50 mL of 1% aqueous bis-hexamethylene triamine solution was prepared. Next, 1 gm of zinc nitrate hexahydrate was dissolved in 50 mL of water separately. These two solutions were mixed and stirred for 1 h. Then, 30 mg of AC was added into the above solution and stirred for 2 h at room temperature. The mixture was poured into a Teflon crucible and kept inside an autoclave at 140 °C for 2 h. After the hydrothermal treatment, the autoclave was allowed to cool naturally. The product was filtered and washed several times with distilled water and ethanol. Finally, as-synthesized ZnO/AC composite was dried at 120 °C for 12 h to improve the crystallinity of ZnO particles. For comparison, pristine ZnO nanorods were also prepared under identical conditions without using AC as reported in our previous work [36]. The fabrication procedure of ZnO nanorods decorated AC derived from lapsi seed stone is schematically depicted in Figure 1.

### 2.4. Characterization

The surface morphology and elemental composition of the samples were studied from field emission scanning electron microscopy (FESEM Hitachi S-7400, Hitachi, Tokyo, Japan) and energy dispersive X-ray spectroscopy. High resolution images were recorded using a transmission electron microscope (TEM, JEOL Ltd., Tokyo, Japan). The crystallographic structure of the synthesized ZnO nanorods on the surface of AC was analyzed by X-ray diffractometer (Rigaku, Japan) with radiation over Bragg angles, ranging from 5 to 90°. The steady-state PL spectroscopy measurement was carried out using a luminescence spectrometer (LS55; Perkin-Elmer Inc., Woodbridge, ON, USA) with a Xe lamp. The N_2_ adsorption–desorption measurements were performed by using the ASAP 2020 Plus system (Micromeritics Instrument Corporation, Norcross, GA, USA).

### 2.5. Photocatalytic Activity Measurement

The photocatalytic activity of the ZnO/AC composite was evaluated by observing the photo-degradation of methylene blue (MB) solution (a typical pollutant in the textile industry) under a mild UV radiation at room temperature. Here, the testing solution into a 100 mL beaker equipped with a light guide (tip of 5 mm diameter) connected to a mercury vapor lamp (OmniCure, EXFO, Richardson, TX, USA) was used for photocatalytic study. The distance between the solution system and the light guide tip was maintained at 5 cm. Then, 20 mg of the photocatalyst was mixed with 20 mL of MB solution which was irradiated under the UV lamp with continuous stirring. During this process, 1 mL of the solution was taken at regular intervals followed by centrifugation to separate the residual catalyst from the solution. Finally, the concentration of MB was observed using a UV-visible spectrophotometer (HP8453, Agilent Technologies, Santa Clara, CA, USA).

## 3. Results and Discussions

**Figure 2:**
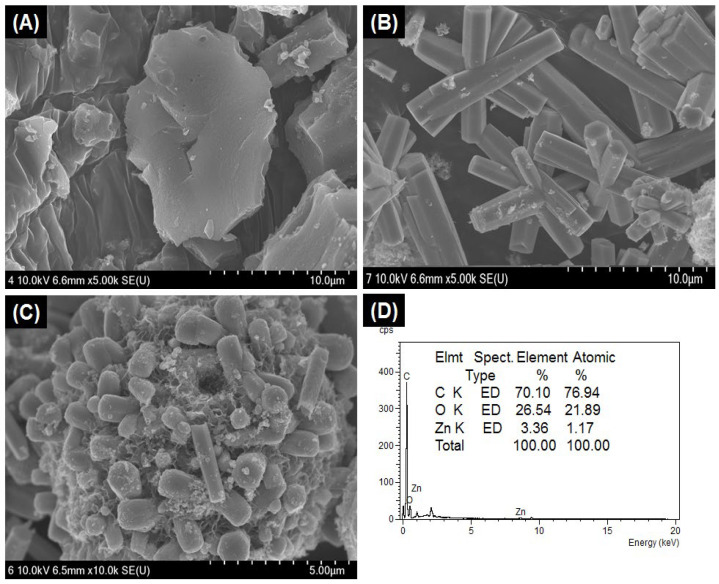
FE-SEM images of activated carbon (**A**), pristine ZnO nanorods (**B**), ZnO/AC composite (**C**), and the EDX of ZnO/AC composite (**D**).

**Figure 3:**
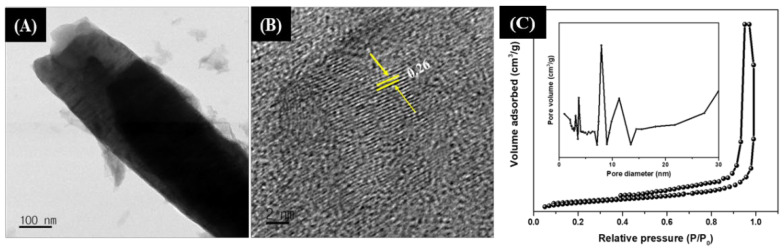
TEM (**A**) and HR-TEM (**B**) of ZnO nanorods grown on AC. Nitrogen adsorption–desorption isotherm and pore size distribution (Inset) of the ZnO/AC composite photocatalyst (**C**).

The morphology of the as-synthesized AC, ZnO, and ZnO/AC composites is shown in Figure 2. The FE-SEM image shows that AC particles are micro-sized flat structures with rough and porous surfaces (Figure 2A). Pristine ZnO particles consist of hexagonal micro-rods, arranged in a bundle-like structure, having an almost equal length and diameter (Figure 2B), whereas the ZnO/AC composite reveals that single hexagonal ZnO nanorods are well developed on the surface of porous AC (Figure 2C). The size of ZnO rods is sufficiently decreased on the surface of AC compared to the pristine ZnO particles. We found that the diameter of the rod in pristine ZnO particles is about 1.2 to 2.1 µm, whereas in composite, the diameter of the rod is reduced to 400–925 nm. Hexagonal heads of micro-rods grown from the center of nuclei in all directions is observed from the FE-SEM image of pristine ZnO particles (Figure 2B), whereas in the ZnO/AC composite, individual rods are grown from the surface of AC. Moreover, the nanosized ZnO rods in the composite still preserved their hexagonal shape. It has been reported that the size and shape of ZnO particles are highly affected by the different physicochemical conditions of the hydrothermal system [37,38,39]. Here, we can consider that the growth of ZnO rods were originated from the center of nuclei [40]. The presence of functionalities on the surface of AC can serve as a nucleation site to grow the ZnO nanorod. When AC was impregnated and stirred in the ZnO precursor solution, the metal ions were uniformly adsorbed on the surface of AC, which acted as nucleation centers for the growth of ZnO nanorods. Therefore, individual rods were independently grown from the surface of AC. Moreover, the dispersion of AC in the same volume of ZnO precursor solution, and nucleation of ZnO at the surface of AC can decrease the concentration of ZnO precursor per unit volume, which consequently decreased the size of the ZnO rods during the hydrothermal process [4,21,22]. Energy dispersive X-ray analysis (EDX) of the as-prepared composite photocatalyst is shown in Figure 2D which further reveals the formation of ZnO particles on the surface of AC. To further incite the morphology of the ZnO rods grown on AC, a TEM analysis was carried out. As given in Figure 3A, a clear rod-like morphology was observed. The observed lattice spacing in HRTEM (Figure 3B) exhibited that the growth of ZnO took place along the [0001] direction, thereby resulting in the formation of hexagonal ZnO nanorods at the surface of AC [41,42,43]. The nitrogen adsorption–desorption isotherm curve and pore size distribution graph (inset) are given in Figure 3C. The surface area of the ZnO/AC composite photocatalyst was 79.86 m^2^g^−1^. The BJH pore size distribution curve showed the mesoporous nature of the AC/ZnO composite.

**Figure 4:**
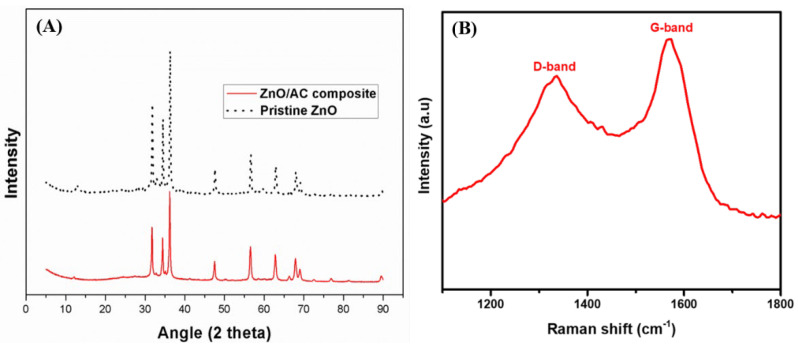
XRD patterns of the pristine ZnO and ZnO/AC composite (**A**) and Raman spectra showing the D and G bands of AC (**B**).

Figure 4A shows the X-ray diffraction (XRD) spectra of the pristine ZnO and ZnO/AC composite photocatalyst. The XRD patterns show the formation of the wurtzite (hexagonal) structure (JCPDS card no. 70-2205) in both the pristine ZnO and ZnO/AC composite. The peaks have been observed at the 2θ angles of 31.9, 34.5, 36.4, 47.7, 56.7, 62.9, 66.5, 68.1, and 69.21°, which are corresponding to the (100), (002), (101), (102), (110), (103), (200), (112), and (201) crystalline planes of ZnO, respectively [22,44]. Furthermore, it can be observed that the XRD intensities of all crystalline planes are stronger in the pristine ZnO nanorods as compared to that of the ZnO/AC composite. The suppression in the intensity of ZnO peaks in the ZnO/AC composite as compared to the pristine ZnO suggests the formation of the composite of ZnO with AC. The carbon peak was not observed in the ZnO/AC composite which might be due to the presence of the high instance peak of ZnO in it. Therefore, the Raman spectra were taken to confirm the presence of carbon. The Raman spectrum showed two peaks at 1347 and 1569 cm^−1^, which can be assigned to the D and G band of carbon (Figure 4B) [32,45].

**Figure 5:**
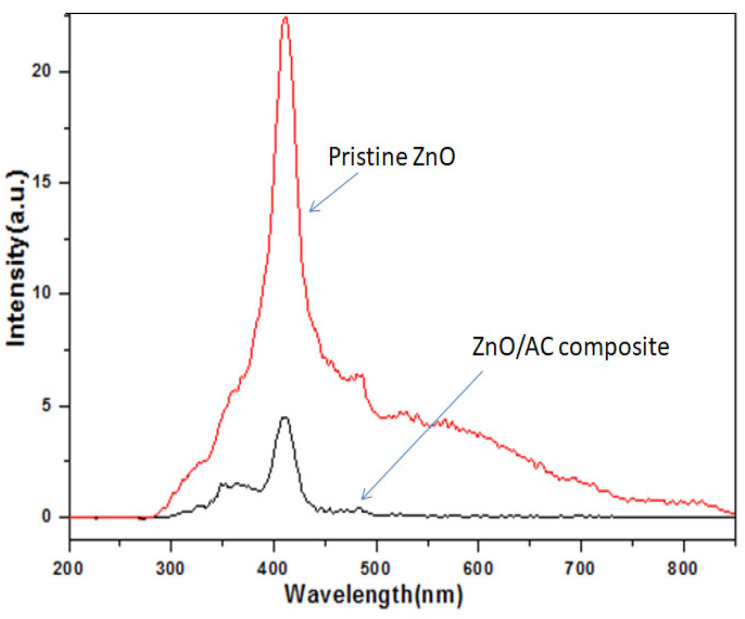
Photoluminescence (PL) emission spectra of the pristine ZnO and ZnO/AC composite.

Photoluminescence (PL) spectra of pristine ZnO and composite photocatalyst are shown in Figure 5. The instance band is observed at the wavelength of ~415 nm, which represents the UV near-band-edge (NBE) emission in pristine ZnO rods [46,47]. This sharp NBE emission peak is due to the recombination of photogenerated electron-hole (e-h) pairs [48]. The ZnO/AC composite showed a decrement in intensity without any shifting of the peak’s position. The rate of recombination between the photogenerated electron-hole pair has thus been lowered in the ZnO/AC composite photocatalyst. This is due to the suppression of the e^−^-h^+^ recombination by the synergistic effect of ZnO/AC. Here, ZnO can act as a good electron donor, whereas carbon materials can act as good electron acceptor. Therefore, the synergistic effect of these two components can reduce the recombination rate and lead to an increase in charge carrier separation. This should enhance the photocatalytic activity of the photocatalyst.

**Figure 6:**
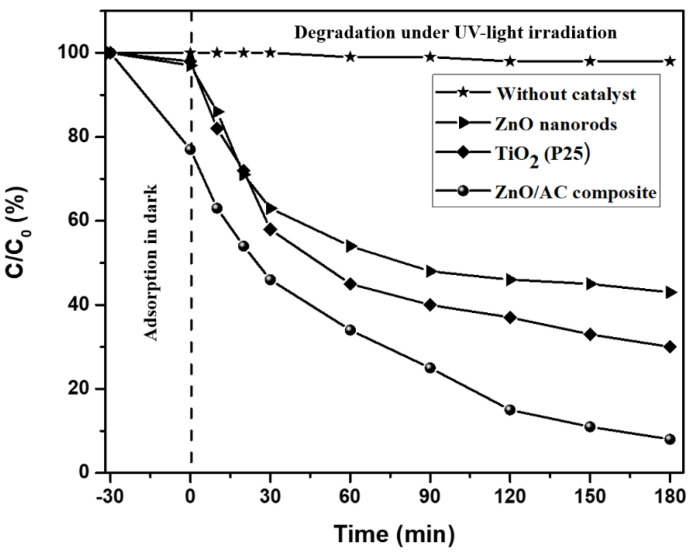
Comparison of photodegradation of MB using different photocatalysts under UV light irradiation.

**Figure 7:**
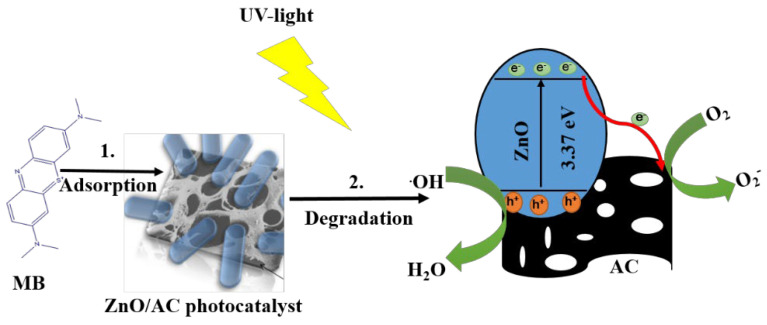
Proposed mechanism of MB removal by simultaneous adsorption and degradation processes.

The photocatalytic performance of the as-synthesized photocatalyst was evaluated by observing the degradation of an aqueous solution of MB. The resulting graph of decreasing in the concentration of MB due to various photocatalysts is shown in Figure 6. The MB may undergo self-degradation under UV-light irradiation. To confirm this, a blank test was performed under UV-light irradiation without using a catalyst. The blank test showed ~2% degradation of MB, which is a negligible amount. The photocatalytic degradation of MB by the pristine ZnO rods was also tested under identical experimental conditions for comparison. We also studied the photocatalytic degradation of MB by using commercially available TiO_2_ NPs (P25). We observed that the ZnO/AC composite exhibited the best photocatalytic performance among the three samples. The photocatalytic results showed that about 95% MB was removed by the ZnO/AC photocatalyst, whereas the pristine ZnO nanorods and P25 showed about 55% and 32% degradation of MB, respectively, after 180 min of UV-light irradiation. This enhanced photocatalytic activity of ZnO/AC compared to pristine ZnO can be attributed to; (i) synergistic effect of ZnO and AC, which can decrease the rate of recombination of electron–hole pairs caused by the trapping of excited electrons from the conduction band of ZnO [23], and (ii) the adsorption of MB molecules by highly porous AC [49,50]. During the first 30 min (dark adsorption test), the ZnO/AC composite showed a steeper slope of the curve compared to the ZnO nanoparticles. It is due to the adsorption property of the ZnO/AC composite. As in the figure, ~33% of the MB dye was adsorbed by AC in the ZnO/AC composite. This finding confirms that in the ZnO/AC, the adsorption and degradation processes take place simultaneously (Figure 7). It is well-known that the photocatalytic activity depends upon the phase structure, particle size, adsorption ability, and e^−^-h^+^ recombination rate [51,52]. From XRD patterns, it is seen that there is no change in the crystal phase of ZnO after loading on the surface of AC. FE-SEM images also show that the size of the ZnO rod is sufficiently decreased without changing their hexagonal structure in ZnO/AC composite. Additionally, it has been reported that the photocatalytic property of the (101) expose crystal plane in ZnO is better than that of the other crystal planes [53]. Therefore, the enhancement in photocatalytic activity of the as-synthesized composite photocatalyst is due to the decreased size of ZnO rods, adsorptive properties of porous AC support, and synergistic effect of ZnO and AC, which led to effective separation of the photo induced e–h pairs.

## 4. Conclusions

In summary, the ZnO nanorods decorated activated carbon composite was prepared for photocatalytic applications. Locally available agricultural waste (lapsi seed stone from Nepal), which also may cause environmental pollution if not disposed of properly, was converted to AC. An effective ZnO/AC composite photocatalyst was prepared by a facile hydrothermal process. Results showed that crystalline ZnO nanorods were well decorated on the AC substrate which reduced the e^−^-h^+^ recombination rate and improved the photocatalytic activity of ZnO/CA composite. The advantages of ZnO/AC in a dye removal system lies in its ability of simultaneous adsorption and dye degradation under UV light irradiation. We believe that the synthetic protocol in this study can be applied for the preparation of AC-based photocatalytic materials from other agricultural wastes.

## References

[1] Pant B., Park M., Park S.-J. (2019). Recent Advances in TiO_2_ Films Prepared by Sol-gel Methods for Photocatalytic Degradation of Organic Pollutants and Antibacterial Activities. Coatings.

[2] Qadri S., Ganoe A., Haik Y. (2009). Removal and recovery of acridine orange from solutions by use of magnetic nanoparticles. J. Hazard. Mater..

[3] Sun B., Li Q., Zheng M., Su G., Lin S., Wu M., Li C., Wang Q., Tao Y., Dai L. (2020). Recent advances in the removal of persistent organic pollutants (POPs) using multifunctional materials: A review. Environ. Pollut..

[4] Pant H.R., Park C.H., Pant B., Tijing L.D., Kim H.Y., Kim C. (2012). Synthesis, characterization, and photocatalytic properties of ZnO nano-flower containing TiO_2_ NPs. Ceram. Int..

[5] Pant B., Park M., Lee J.H., Kim H.-Y., Park S.-J. (2017). Novel magnetically separable silver-iron oxide nanoparticles decorated graphitic carbon nitride nano-sheets: A multifunctional photocatalyst via one-step hydrothermal process. J. Colloid Interface Sci..

[6] Haider A.J., Anbari R.A., Sami H.M., Haider M.J. (2019). Photocatalytic Activity of Nickel Oxide. J. Mater. Res. Technol..

[7] Serpone N., Emeline A.V. (2012). Semiconductor Photocatalysis—Past, Present, and Future Outlook. J. Phys. Chem. Lett..

[8] Rajendran S., Khan M.M., Gupta V.K., Mosquera E., Gracia F., Narayanan V., Stephen A. (2015). ZnO/Ag/CdO nanocomposite for visible light-induced photocatalytic degradation of industrial textile effluents. J. Colloid Interface Sci..

[9] Ong C.B., Ng L.Y., Mohammad A.W. (2018). A review of ZnO nanoparticles as solar photocatalysts: Synthesis, mechanisms and applications. Renew. Sustain. Energy Rev..

[10] Johar M.A., Afzal R.A., Alazba A.A., Manzoor U. (2015). Photocatalysis and Bandgap Engineering Using ZnO Nanocomposites. Adv. Mater. Sci. Eng..

[11] Lee K.M., Lai C.W., Ngai K.S., Juan J.C. (2016). Recent developments of zinc oxide based photocatalyst in water treatment technology: A review. Water Res..

[12] Pant B., Ojha G.P., Kim H.-Y., Park M., Park S.-J. (2019). Fly-ash-incorporated electrospun zinc oxide nanofibers: Potential material for environmental remediation. Environ. Pollut..

[13] Kaviya S., Rajendran S., Naushad M., Ponce L.C., Lichtfouse E. (2020). Evolution of ZnO-Based Photocatalyst for the Degradation of Pollutants. Green Photocatalysts for Energy and Environmental Process.

[14] Xu C., Anusuyadevi P.R., Aymonier C., Luque R., Marre S. (2019). Nanostructured materials for photocatalysis. Chem. Soc. Rev..

[15] Pant B., Barakat N.A., Pant H.R., Park M., Saud P.S., Kim J.-W., Kim H.-Y. (2014). Synthesis and photocatalytic activities of CdS/TiO_2_ nanoparticles supported on carbon nanofibers for high efficient adsorption and simultaneous decomposition of organic dyes. J. Colloid Interface Sci..

[16] Wang N., Sun C., Zhao Y., Zhou S., Chen P., Jiang L. (2008). Fabrication of three-dimensional ZnO/TiO_2_ heteroarchitectures via a solution process. J. Mater. Chem..

[17] Pant H.R., Pant B., Kim H.J., Amarjargal A., Park C.H., Tijing L.D., Kim E.K., Kim C. (2013). A green and facile one-pot synthesis of Ag–ZnO/RGO nanocomposite with effective photocatalytic activity for removal of organic pollutants. Ceram. Int..

[18] Azmina M.S., Nor R.M., Rafaie H.A., Razak N.S.A., Sani S.F.A., Osman Z. (2017). Enhanced photocatalytic activity of ZnO nanoparticles grown on porous silica microparticles. Appl. Nanosci..

[19] Pant B., Park M., Park S.-J. (2019). MoS2/CdS/TiO_2_ ternary composite incorporated into carbon nanofibers for the removal of organic pollutants from water. Inorg. Chem. Commun..

[20] Pant B., Park M., Kim H.-Y., Park S.-J. (2016). Ag-ZnO photocatalyst anchored on carbon nanofibers: Synthesis, characterization, and photocatalytic activities. Synth. Met..

[21] Pant B., Pant H.R., Barakat N.A., Park M., Jeon K., Choi Y., Kim H.-Y. (2013). Carbon nanofibers decorated with binary semiconductor (TiO_2_ /ZnO) nanocomposites for the effective removal of organic pollutants and the enhancement of antibacterial activities. Ceram. Int..

[22] Pant B., Ojha G.P., Kuk Y.-S., Kwon O.H., Park Y.W., Park M. (2020). Synthesis and Characterization of ZnO-TiO_2_/Carbon Fiber Composite with Enhanced Photocatalytic Properties. Nanomaterials.

[23] Mu J., Shao C., Guo Z., Zhang Z., Zhang M., Zhang P., Chen B., Liu Y. (2011). High Photocatalytic Activity of ZnO−Carbon Nanofiber Heteroarchitectures. ACS Appl. Mater. Interfaces.

[24] Gu C., Xiong S., Zhong Z., Wang Y., Xing W. (2017). A promising carbon fiber-based photocatalyst with hierarchical structure for dye degradation. RSC Adv..

[25] Ge J., Zhang Y., Park S.-J. (2019). Recent Advances in Carbonaceous Photocatalysts with Enhanced Photocatalytic Performances: A Mini Review. Materials.

[26] Dąbrowski A., Podkościelny P., Hubicki Z., Barczak M. (2005). Adsorption of phenolic compounds by activated carbon—A critical review. Chemosphere.

[27] Martins A., Nunes N. (2014). Adsorption of a Textile Dye on Commercial Activated Carbon: A Simple Experiment To Explore the Role of Surface Chemistry and Ionic Strength. J. Chem. Educ..

[28] Nasrollahzadeh M.S., Hadavifar M., Ghasemi S.S., Chamjangali M.A. (2018). Synthesis of ZnO nanostructure using activated carbon for photocatalytic degradation of methyl orange from aqueous solutions. Appl. Water Sci..

[29] Park N.-K., Lee Y.J., Han G.B., Ryu S.O., Lee T.J., Chang C.H., Han G.Y. (2008). Synthesis of various zinc oxide nanostructures with zinc acetate and activated carbon by a matrix-assisted method. Colloids Surf. A Physicochem. Eng. Asp..

[30] Muthirulan P., Meenakshisundararam M., Kannan N. (2013). Beneficial role of ZnO photocatalyst supported with porous activated carbon for the mineralization of alizarin cyanin green dye in aqueous solution. J. Adv. Res..

[31] Hassan M.F., Sabri M.A., Fazal H., Hafeez A., Shezad N., Hussain M. (2020). Recent trends in activated carbon fibers production from various precursors and applications—A comparative review. J. Anal. Appl. Pyrolysis.

[32] Awasthi G.P., Bhattarai D.P., Maharjan B., Kim K.-S., Park C.H., Kim C.S. (2019). Synthesis and characterizations of activated carbon from Wisteria sinensis seeds biomass for energy storage applications. J. Ind. Eng. Chem..

[33] El Nemr A., Abdelwahab O., El-Sikaily A., Khaled A. (2009). Removal of direct blue-86 from aqueous solution by new activated carbon developed from orange peel. J. Hazard. Mater..

[34] Ip A., Barford J.P., McKay G. (2008). Production and comparison of high surface area bamboo derived active carbons. Bioresour. Technol..

[35] Shrestha R.M., Yadav A.P., Pokharel P.B., Pradhananga R.R. (2012). Preparation and Characterization of Activated Carbon from Lapsi (Choerospondias axillaris) Seed Stone by Chemical Activation with Phosphoric Acid. Res. J. Chem. Sci..

[36] Pant H.R., Pant B., Sharma R.K., Amarjargal A., Kim H.J., Park C.H., Tijing L.D., Kim C.S. (2013). Antibacterial and photocatalytic properties of Ag/TiO_2_/ZnO nano-flowers prepared by facile one-pot hydrothermal process. Ceram. Int..

[37] Wang S., Gao M., Ma B., Xi M., Kong F. (2020). Size-dependent effects of ZnO nanoparticles on performance, microbial enzymatic activity and extracellular polymeric substances in sequencing batch reactor. Environ. Pollut..

[38] Wang X., Zhang Q., Wan Q., Dai G., Zhou C., Zou B. (2011). Controllable ZnO Architectures by Ethanolamine-Assisted Hydrothermal Reaction for Enhanced Photocatalytic Activity. J. Phys. Chem. C.

[39] Wasly H.S., El-Sadek M.S.A., Henini M. (2018). Influence of reaction time and synthesis temperature on the physical properties of ZnO nanoparticles synthesized by the hydrothermal method. Appl. Phys. A.

[40] Xu C., Liu Z., Liu S., Wang G. (2003). Growth of hexagonal ZnO nanowires and nanowhiskers. Scr. Mater..

[41] Luo S., Liu C., Zhou S., Li W., Ma C., Liu S., Yin W., Heeres H.J., Zheng W., Seshan K. (2020). ZnO nanorod arrays assembled on activated carbon fibers for photocatalytic degradation: Characteristics and synergistic effects. Chemosphere.

[42] Ding J., Fang X., Yang R., Kan B., Li X., Yuan N. (2014). Transformation of ZnO polycrystalline sheets into hexagon-like mesocrystalline ZnO rods (tubes) under ultrasonic vibration. Nanoscale Res. Lett..

[43] Saravanan A., Huang B.-R., Kathiravan D., Prasannan A. (2017). Natural Biowaste-Cocoon-Derived Granular Activated Carbon-Coated ZnO Nanorods: A Simple Route To Synthesizing a Core–Shell Structure and Its Highly Enhanced UV and Hydrogen Sensing Properties. ACS Appl. Mater. Interfaces.

[44] Muhammad W., Ullah N., Haroon M., Abbasi B.H. (2019). Optical, morphological and biological analysis of zinc oxide nanoparticles (ZnO NPs) using Papaver somniferum L.. RSC Adv..

[45] Wang Y., Alsmeyer D.C., McCreery R.L. (1990). Raman spectroscopy of carbon materials: Structural basis of observed spectra. Chem. Mater..

[46] Lim J., Shin K., Kim H.W., Lee C. (2004). Effect of annealing on the photoluminescence characteristics of ZnO thin films grown on the sapphire substrate by atomic layer epitaxy. Mater. Sci. Eng. B.

[47] Raji R., Gopchandran K. (2017). ZnO nanostructures with tunable visible luminescence: Effects of kinetics of chemical reduction and annealing. J. Sci. Adv. Mater. Devices.

[48] Yang J., Zheng J., Zhai H., Yang X., Yang L., Liu Y., Lang J., Gao M. (2010). Oriented growth of ZnO nanostructures on different substrates via a hydrothermal method. J. Alloy. Compd..

[49] Pathania D., Sharma S., Singh P. (2017). Removal of methylene blue by adsorption onto activated carbon developed from Ficus carica bast. Arab. J. Chem..

[50] Pereira M.F.R., Soares S.F., Órfão J.J.M., Figueiredo J.L. (2003). Adsorption of dyes on activated carbons: Influence of surface chemical groups. Carbon.

[51] Chen S., Zhao W., Liu W., Zhang S. (2008). Preparation, characterization and activity evaluation of p–n junction photocatalyst p-ZnO/n-TiO_2_. Appl. Surf. Sci..

[52] Yang X., Wang D. (2018). Photocatalysis: From Fundamental Principles to Materials and Applications. ACS Appl. Energy Mater..

[53] Zhou T., Hu M., He J., Xie R., An C., Li C., Luo J. (2019). Enhanced catalytic performance of zinc oxide nanorods with crystal plane control. CrystEngComm.

